# The Means of Promoting and Preserving Health

**Published:** 1842-04

**Authors:** 


Art. VI.—*The Means of Promoting and Preserving Health.* By T. Hodgkin, m.d. Second Edition, with additions.—*London,* 1841. 8vo, pp. 480.

In the Second Number of this Journal, for April, 1836, we noticed at considerable length the first edition of this little work, and bestowed on it the high commendation which its merits claimed. The present edition, which has been long wanted, is greatly improved by valuable and extensive additions to each lecture. Many of these additions have been prepared with a less exclusive view to the working classes of society than the original lectures, and hence the book, in its present form, will be found better calculated for general perusal, and consequently be of more extensive usefulness. To all classes of readers we earnestly recommend this little volume, as fraught with information which it behoves every well-wisher of his fellow-men and of his country to be acquainted with.

